# The Relationship between Affiliate Stigma in Parents of Children with Autism Spectrum Disorder and Their Children’s Activity Participation

**DOI:** 10.3390/ijerph17051799

**Published:** 2020-03-10

**Authors:** Carmen K. M. Ng, Stephen H. F. Lam, Sally T. K. Tsang, Cheong M. C. Yuen, Chi-Wen Chien

**Affiliations:** Department of Rehabilitation Sciences, The Hong Kong Polytechnic University, Hung Hom, Kowloon, Hong Kong (SAR), China

**Keywords:** autism spectrum disorder, parents, affiliate stigma, activity participation, young children’s participation and environment measure, preschool-aged children

## Abstract

Children with autism spectrum disorder (ASD) are reported to participate less in everyday activities, and their parents face stigma on account of having a child with ASD, which they often internalize as affiliate stigma. Studies have examined the impact of affiliate stigma on parents’ psychological well-being and social behaviors, but little is known about how affiliate stigma impacts their children’s activity participation. This study aimed to investigate the relationship between parents’ affiliate stigma and activity participation of their children with ASD. Sixty-three parents of children with ASD (aged 2–6 years) were recruited. They completed questionnaires, which captured affiliate stigma, their child’s participation (frequency and involvement) in home, preschool, and community activities, and demographic characteristics. Results indicated that these parents had a moderate level of affiliate stigma, which did not correlate with the frequency of their children’s participation in activities. However, the parents’ affiliate stigma was found to have negative impacts on their children’s involvement in overall community participation and participation in one particular activity at home. The findings highlight the importance of destigmatization of parents of children with ASD in order to promote their children’s participation in community activities.

## 1. Introduction

Autism spectrum disorder (ASD) encompasses a broad group of neurodevelopmental disorders that result in deficits in social communication and the presence of stereotypic and repetitive behaviors (e.g., flapping, finger-gazing, and self-stimulation) [1]. Due to the communicative and behavioral problems, children with ASD are reported to have difficulties in self-care, social interaction, and developing friendships with others [2]. In preschool-aged children with ASD, challenges particularly arise from the vulnerabilities linked to newly emerging cognitive, speech, motor, and social skills [3]. Thus, children during this critical developmental stage are reliant mostly on their parents for participation in everyday activities [4,5].

Participation has been recognized as an important outcome and an ultimate goal for rehabilitation services in children with disabilities including ASD [6,7]. According to the World Health Organization [8], participation refers to an individual’s involvement in life situations and, for children, it usually takes place in a social context involving family members, peers, or other adults [9,10]. Participation in everyday activities provides learning opportunities for children, which in turn benefits their growth and development [6,11,12]. King and her colleagues [11] found that activity participation contributed to children’s well-being and, through engaging in activities with others, children also developed social skills and communication abilities, which helped form mutual relationships. With respect to psychological aspects, children may additionally develop self-identity and self-esteem when interacting with their peers or adults during activity participation [9]. Despite the importance of participation, preschool-aged children with ASD have been reported to participate less in educational, recreational, community, and social activities, compared to their typically developing peers [5,13,14].

Recent studies have investigated factors that may support or hinder activity participation of children with ASD while considering intervention planning and service provisions [15,16,17,18,19]. There seems to be a consensus about the extent to which children with ASD take part in activities that entail social interaction (e.g., community activities), which may be influenced by the family environment [20,21]. In fact, for preschool-aged children with ASD, the family plays a crucial role in determining the type and number of activities in which the children will be given opportunities for participation [22]. For example, Lam and her colleagues [23] found that parents’ willingness to take their child with ASD to engage in community activities was associated with the degree of difficulty and negative emotions of the children as perceived by the parents. However, this association was not observed in children with typical development. In a study by LaVesser and Berg [5], barriers to participation for children with ASD from parental perspectives were investigated. The children’s behavioral problems appeared to increase parents’ stress and burden and in turn, deterred them from offering their children with ASD opportunities to participate in community activities. This implies that the parents may experience a certain degree of stigmatization that leads them to develop negative emotions, withdraw from social situations, and provide limited opportunities for themselves or their children to positively interact with others. It is possible that the stigma experienced by the parents turns into a barrier for activity participation for their children with ASD.

According to a recent review [24], parents of children with ASD are often reported to encounter varied forms of stigmatization on account of their children having ASD. Stigma in general is a set of prejudicial attitudes, discriminatory behaviors, stereotypes, and biased social structures upheld by a sizeable group about a discredited subgroup [25]. Stigma not only affects the discredited individuals themselves, but it can also impact caregivers and family members of the stigmatized individuals. In the case of children with ASD, parents may perceive courtesy stigma (also known as family or associative stigma) from the general public toward themselves due to people’s misconceptions about the lack of parental control or discipline of their child’s behavioral problems. Through repeated exposure to courtesy stigma, parents may develop affiliate stigma (conceptualized as self-stigma of family members [26]) by which they may feel upset and helpless about the affiliation with their child with ASD and perceive negative emotions and cognitions toward themselves [27]. Affiliate stigma could lead the parents to react behaviorally by hiding their children’s condition from others, withdrawing from social relations, or alienating themselves from the stigmatized individuals to avoid association [28,29]. There are a few studies that have reported a high level of affiliate stigma in the parents of children with ASD [27,30,31,32,33]. Affiliate stigma is also found to negatively affect psychological well-being in these parents, leading to a reduced quality of life [24,27,30].

As outlined above, most studies focused on separately investigating either the affiliate stigma in parents of children with ASD or the activity participation of their children. However, little is known about whether parents’ affiliate stigma would limit activity participation of their children with ASD, especially at the preschool age during which parents play a crucial role in arranging participation opportunities for their child [5,22]. To fill the knowledge gap, the current study aimed to investigate whether the parents’ affiliate stigma was related to activity participation of their child, aged 26 years with ASD. Based on existing evidence [5,23], we hypothesized that affiliate stigma in parents of preschool-aged children with ASD would have negative associations with their child’s participation in community activities, which involve social interaction with other children and adults, but not in home or daycare/preschool activities that are solitarily or with assistance from teachers who are familiar to children.

## 2. Materials and Methods

### 2.1. Participants

Children with ASD and their parents were recruited from four early education and training centers and three special child-care centers from a local nongovernment organization in Hong Kong. These centers mainly provided assessment, training, guidance, and family support to preschool-aged children with diverse special needs. Parents who participated in this study needed to meet the following inclusion criteria: (1) their child was aged between 2–6 years old, (2) their child was diagnosed with ASD by pediatricians in child assessment centers or by psychologists in hospitals, and (3) they were able to read and write Chinese. Children were excluded if they had serious illnesses (e.g., blindness) or physical impairments (e.g., amputation), as their participation patterns could be different from those of children without medical comorbid diagnoses/disabilities.

### 2.2. Instruments

#### 2.2.1. Participation Measure

Young Children’s Participation and Environment Measure (YC-PEM) [34] is a caregiver-report questionnaire which evaluates the extent of activity participation of children aged 0 to 5 years in three types of environmental settings (home, daycare/preschool, and community). The caregiver needs to complete 27 items across each setting: home (13 items), daycare/preschool (3 items), and community (11 items). The types of activities in which children participate at home include basic self-care routines, household, interactive and organized play, as well as socializing with friends and family. The types of daycare/preschool activities include educational programs related to group learning, socializing with friends, as well as field activities and school events. For community activities, the activity types include outings, class and group activities, community-related activities, and recreational/leisure activities.

For each activity, caregivers are asked to evaluate three dimensions of child participation: a) the frequency of participation, using an 8-point Likert scale (never = 0 and once or more each day = 7); b) the degree of involvement, using a 5-point Likert scale (not very involved = 1 and very involved = 5); and c) whether the caregivers want a change in their child’s participation (yes or no). If the caregivers desire a change, they are asked to specify the type(s) of change desired (do more often, do less often, be more interactive, be more helpful, and participate in a broader variety of activities). Three types of participation summary scores (frequency, involvement, and desire for change) can be produced for each of the three settings in YC-PEM. The score calculation is detailed in Khetani et al. [34]. Higher scores indicate higher frequency, involvement, and a greater desire for change in the child’s participation. In this study, we analyzed the frequency and involvement dimensions because they are two important aspects representing children’ participation patterns [35]. The parents’ desire-for-change dimension was not included in the analysis owing to the substantial ceiling effects of 48%–92%, which were revealed in the study sample. The Chinese version of YC-PEM [36] was used in this study to capture children’s participation specifically in Hong Kong. This Chinese version was translated from the original YC-PEM and adapted into the local context by following the guidelines provided by the YC-PEM’s authors and other researchers [37,38]. Internal consistency of the Chinese YC-PEM was acceptable for the frequency dimension (Cronbach’s alpha = 0.42–0.70) and for the involvement dimension (0.73–0.77), which was comparable to the original YC-PEM [34].

#### 2.2.2. Affiliate Stigma Measure

This study used the Chinese version of the affiliate stigma scale (ASS), which was developed by Mark and Cheung [26] originally to measure affiliate stigma in caregivers of people with intellectual disability and was subsequently adapted and used in caregivers of children with ASD by the same research team [27]. This scale consists of 22 items scored on a 4-point Likert scale (strongly disagree = 1 and strongly agree = 4). Mean scores can be generated by averaging the scores of the 22 items, with higher scores indicating higher levels of affiliate stigma. ASS has been reported to demonstrate excellent internal consistency (Cronbach’s alpha = 0.94) as per a previous study by Mak and Kwok [27].

#### 2.2.3. Demographic Information

Demographic characteristics about the parents and children were obtained using a questionnaire which was specifically designed for this study. For the children, we collected gender, age, the types of comorbidity which the child had in addition to ASD, and the number of siblings the child had in the family. For the parents, we collected his/her role in relation to the child, age, the highest educational qualification, and monthly family income. We did not obtain information about the participants’ ethnicity as 92% of people living in Hong Kong are Chinese [39].

### 2.3. Procedure

All participants gave their informed consent for inclusion before they participated in the study. The study was conducted in accordance with the Declaration of Helsinki. Ethical approval was granted by the ethical review committee at The Hong Kong Polytechnic University (Reference number: HSEARS20180111006). Invitation packets for parents’ participation in the study were sent to each of the participating early education and training centers and special child-care centers. These invitation packets contained information sheets, consent forms, research questionnaires (YC-PEM, ASS, and demographic questionnaire), and return prepaid envelope. Speech therapists in the participating centers helped to distribute the invitation packets to eligible participants of interest. Parents of each child were asked to complete the written consent and research questionnaires and return them to the centers or to us by post. A supermarket voucher of HKD 50 was given to all the participants who completed the questionnaires.

### 2.4. Data Analysis

All data were inputted into and analyzed using the Statistical Package for Social Science (SPSS) version 23 (SPSS Inc., Chicago, IL, USA) Data were checked for normality by the means of histograms and Kolmogorov-Smirnov tests. We found that two participation frequency variables (the summary scores in the home and daycare/preschool settings) and four demographic variables did not present a normal distribution. To enable parametric analyses, these two participation variables were log-transformed, whereas the four demographic variables were collapsed into dichotomous categories.

To investigate the extent to which the affiliate stigma in parents of children with ASD was associated with their children’s participation, and by taking into account the influence of demographic variables on participation, six hierarchical multiple linear regression models were performed. Three models focused on participation frequency in each of the home, daycare/preschool, and community settings, and the other three models targeted participation involvement.

To accommodate for the small sample size of this study, we performed the analyses in three steps. First, five demographic variables that might influence children’s participation (based on literature [11,20,40]) were examined univariably. These variables were presence of comorbidity, number of siblings, monthly family income, and parents’ age and educational qualification. Only the variables that showed significance at the *p* < 0.05 level in each of the univariate models were considered for inclusion in corresponding regression models in the next step. In addition, we included child age in all the models of the next step, given that age has been frequently reported as an important predictor of children’s participation [11,16]. However, only a few girls (*n* = 9) were recruited in the current study, so child gender was neither tested nor included in any regression models.

For the next step, hierarchical multiple linear regression analyses were conducted. Each of the two participation summary scores for the three settings served as the dependent variable. Child age and other demographic variables (if significant in the previous step) were included as control variables in the first block of the models. In the second block of the models, the parents’ affiliate stigma served as the independent variable. The significance level was set at *p* < 0.05. Essential assumptions (i.e., multicollinearity, homoscedasticity, normality, and linearity) for multiple regression analyses were verified (using the correlation matrix for the independent variables, variance inflation factors, and residual analyses) [41].

For the last step, we validated all the hierarchical multiple linear regression models using bootstrapping methods, in which 2000 repeated samples with the same size as the present sample were drawn at random (with replacement). Bootstrapping estimates and confidence intervals were then produced using the 2000 replications for comparison [42]. In addition, we performed Spearman’s rho correlation to examine the association between parents’ affiliate stigma and children’s participation in each activity. Nonparametric statistics were used for the item-level analyses as most of the participation item scores in the current sample were found not to have a normal distribution. 

## 3. Results

Of 160 research packets distributed, a total of 15 fathers and 48 mothers participated in the study (response rate: 39.38%). The children with ASD had a mean age of 5.45 years with a standard deviation (SD) of 0.96 years. Most of the children had more than one sibling (60.3%). About two-thirds of the children were reported to have a comorbidity other than ASD, with 57.1% having developmental delays. The responding parents’ mean age was 39.07 years old (SD = 6.03), and approximately one-third of the parents completed undergraduate or higher degrees. Table 1 and Table 2 show the demographic characteristics of the children and their parents, respectively.

For the children’s participation as measured by YC-PEM, they participated relatively frequently in activities at home (median = 4.69, interquartile range = 4.23–5.46) and daycare/preschool activities (median = 5.00, interquartile range = 3.67–5.33), but not in community activities (mean = 3.09, SD = 0.70). Their participation involvement in these activities was similar across home (mean = 3.52, SD = 0.68), daycare/preschool (mean = 3.25, SD = 1.44), and community settings (mean = 3.41, SD = 0.82). In addition, the mean ASS scores of the parents was 2.21 (SD = 0.48).

Table 3 summarizes the demographic variables that were significantly associated with the children’s participation in each of the univariate regression models across the three settings (home, daycare/preschool, and community). For the frequency dimension, two significant associations were found between the respondents’ age and children’s participation in activities at home and between the respondents’ educational level and children’s participation in daycare/preschool activities. For the involvement dimension, the presence of a comorbidity other than ASD demonstrated significant associations with participation in home and community activities. Also, the respondents’ educational level was significantly associated with the children’s participation in activities at home.

Table 4 shows the associations between the ASS mean scores and YC-PEM summary scores, in the hierarchical regression models, which controlled for one to three demographic variables that were identified as significant in the above univariate models. The parents’ affiliate stigma was unrelated to the frequency of children’s participation in home, daycare/preschool, and community activities. Similarly, the parents’ affiliate stigma was also unrelated to the children’s participation involvement in home and daycare/preschool activities. However, it was negatively related to the children’s participation involvement in community activities (β = −0.329, *p* = 0.009). The bootstrapping methods confirmed similar results in each model. Particularly, the parents’ affiliate stigma was found not to include zero in the nonstandardized estimates within the 95% bias-corrected accelerated confidence intervals in the model of the community participation involvement. This indicated that parents’ affiliate stigma was identified as significant in the 2000 bootstrapping resamples.

Table 5 shows Spearman’s rho correlation coefficients between the ASS mean scores and item scores of each activity in YC-PEM. The children’s participation involvement in two specific activities (getting clean at home and organized physical activities in the community) were found to significantly correlate with the parents’ affiliate stigma (−0.289, *p* < 0.05 and −0.453, *p* < 0.01). There were no significant correlations between the frequency of children’s participation in any activity and the parents’ affiliate stigma. 

## 4. Discussion

The present study is the first to investigate the relationship between parents’ affiliate stigma and participation of their preschool-aged children with ASD in home, daycare/preschool, and community activities. We found that these parents’ mean ASS scores were above the midpoint of the scale, indicating that they were suffering from internalized stigma to a moderate extent. However, the findings of this study showed that, as a whole, the parents’ affiliate stigma did not correlate with the frequency and involvement of their children’s participation (except for involvement in community activities). There were also a few significant correlations between the parents’ affiliate stigma and children’s participation involvement in one activity both at home and in the community. These findings suggest that parents’ affiliate stigma might not systematically affect the frequency of their children’s participation but impact their involvement when taking part in community activities. The findings of this study may be helpful for service providers to design and plan interventions that promote the involvement of community participation for preschool-aged children with ASD by attempting to alleviate the affiliate stigma their parents experience.

The level of affiliate stigma reported by the parents of children with ASD in this study was relatively high. The findings are similar to previous studies [27,43] that included parents of children with ASD in the same region (i.e., Hong Kong), although the children in previous studies were older (mean age, 9.77 and 9.82 years) than those in our study (mean age, 5.45 years). This indicates that parents might have been internalizing stigma from the time their children with ASD were at the preschool stage. Moreover, parents in Hong Kong tended to experience higher affiliate stigma in comparison with parents in other countries [31,33]. Similar trends are also observed in parents of preschool-aged children. For example, Patra and Kumar Patro [44], who studied parents of Indian children with ASD aged 3–7 years, reported a mean ASS score of 2.07, which is slightly lower than that in our study (2.20). The difference may stem from the fact that stigma is influenced by culture [27]. Culture in Hong Kong originated from China and is based on collectivism, which is characterized by a close linkage between individuals with a strong sense of obligation to the group and not the individual [45,46]. People who deviate from social norms are likely to be stigmatized within collectivist cultures. As such, people may be prone to considering young children with disabilities as “bad seeds” and a disgrace to their families [47]. Given the influence of culture on stigma, parents in Hong Kong may be exposed to higher levels of courtesy stigma, which in turn develops into a higher level of affiliate stigma as their children with ASD grow up [24,27]. Future studies are warranted to investigate the influence of Hong Kong parents’ collectivistic values on perceived stigma associated with their children with ASD.

The results indicate an insignificant relationship between the parents’ affiliate stigma and the frequency of their children’s participation, which does not fully match our hypotheses that affiliate stigma might lead to reduced participation in community activities. Our findings, however, may be reflected in a study by Lam et al. [23], in which the parents of preschoolers with ASD showed high levels of negative emotion and low willingness to take their children out, and these two variables were found to have no association with the participation frequency in community activities. One reason for the similarity in findings between our study and that of Lam et al. could be that participation in some community activities is habitual (or obligatory) for the children and families. Parents who experience affiliate stigma may still have to take their child out for extracurricular classes in the community or along with them for shopping at grocery stores if there is no one to look after the child. Likewise, the habituality (or necessity) of participation especially for preschool-aged children is also pertinent to home (e.g., mealtime or homework) and daycare/preschool activities (e.g., classroom activities), in terms of the frequency. This may explain why the frequency of children’s participation in activities across all the settings was not associated with the parents’ affiliate stigma.

Different from the above findings regarding participation frequency, the present study found a significant relationship between parents’ affiliate stigma and their children’s participation involvement in community activities based on the regression analysis results. Specifically, the correlation analysis identified that the children’s involvement in one community activity (organized physical activities) and one home activity (getting clean) was associated negatively with the parents’ affiliate stigma. Involvement has been recently constructed as one of two key dimensions of participation in addition to frequency [35]. It is conceptualized as “the experience of participation” which may include elements of motivation, persistence, social connection, or level of affect [35,48]. Thus, internalized stigma perceived by parents may hinder their children’s involvement when engaging in activities, and this impact may be specific to the nature of certain activities. For example, getting clean (e.g., washing or wiping hands and face) has been found to be one of the important activities at home in which preschool-aged children with ASD participated most frequently, both in our study and that of LaVesser and Berg [5]. This activity may be perceived by the parents as important when compared to other activities at home. We further speculate that, if children with ASD have problems in getting their hand/face/body clean which may lead to poor personal hygiene and illness (e.g., diarrhea), the parents may be stigmatized by other family members at home. These parents may then blame themselves for causing the problems and develop certain levels of affiliate stigma [27]. As a result, parents with this self-stigma may provide excessive assistance in keeping their children clean to ensure the hygiene or health, leading to a decrease in their children’s involvement in this activity.

On the other hand, participation in community activities such as organized physical activities require high social demand from children to interact with other people. It could thus be difficult for children with ASD who exhibit bizarre or challenging behaviors to be involved fully in community activities [5]. Especially children with ASD may not be easily identified by their physical appearance. When children with ASD display any of the socially unacceptable behaviors, people in the community may stigmatize their parents for not exerting control over the children’s behaviors. In turn, these parents may develop high levels of affiliate stigma due to a self-perceived inability [27]. Therefore, the parents of children with ASD may feel embarrassed or ashamed and choose to escape or intervene immediately, followed by a reduced involvement of their children. This may explain the relationship between the parents’ affiliate stigma and their children’s involvement when engaging in community activities.

The present study has several limitations. First, this study made use of convenience sampling, which might not have included parents who suffered from severe affiliate stigma. Furthermore, we excluded children with ASD who had serious illnesses or physical impairments, whose parents might also experience severe affiliate stigma. The restricted range of the severity of parents’ affiliate stigma could lead to a lack of or weak relationship between parents’ affiliate stigma and their children’s participation frequency. Second, the sample size of this study was small. Thus, the statistical power might not be adequate enough to detect significant correlations between affiliate stigma and participation. Third, there was a gender bias toward boys with ASD (85.7%) in the sample. The male-to-female ratio was higher than the estimated ratio of 3:1 in a recent meta-analysis [49], indicating that the sample of this study was not representative of the larger population. While the bootstrapping methods were used in the present study to replicate the results, future studies are needed to confirm the study’s findings by recruiting a larger sample and including more parents who experience severe affiliate stigma, as well as more girls with ASD. Studies that compare the effect of having medical comorbid diagnoses/disabilities in children with ASD on the relationship between their participation and parents’ affiliate stigma are also warranted.

In spite of the above limitations, this study has important implications of interventions for families and their children with ASD. For example, a reduction in parents’ internalized stigma may be included as part of participation-focused interventions with children. This could be achieved through the provision of parenting/coping skills or establishment of mutual support groups to enhance their sense of control and social support [27], which in turn may reduce their negative emotion or excessive assistance in their children’s activity participation. Also, collaborations with nongovernment organizations and government sectors may be necessary to provide public education and exposure to ASD [24,30]. Public stigma towards children with ASD and their families could be reduced accordingly and these children may be involved more in community activities. In addition, the findings of the present study provide a basis for further research to study the impact of affiliate stigma on children’s activity participation by taking into account other possible factors/mediators in the testing models. 

## 5. Conclusions

This study adds knowledge about the relationship between parents’ affiliate stigma and their preschool-aged children’s participation in home, daycare/preschool, and community activities. Parents’ affiliate stigma was found not to correlate with the frequency of their children’s participation but led to reduced involvement of the children when engaging in community activities. Furthermore, these parents exhibited a moderate level of affiliate stigma, which requires special attention. To promote the children’s participation, especially their involvement in community activities, interventions and resources that support parents who are taking care of preschool-aged children with ASD are needed to help alleviate their internalized stigma.

## Figures and Tables

**Table 1:** Characteristics of children in the study.

Characteristics.	Value
Age, years, mean ± SD	5.45 ± 0.96
Gender, *n* (%)	
Female	9 (14.3)
Male	54 (85.7)
Number of siblings, * *n* (%)	
0	24 (38.1)
1	31 (49.2)
2 or more	7 (11.1)
Parent-reported diagnoses, *n* (%)	
Only Autism Spectrum Disorder	21 (33.3)
Autism Spectrum Disorder with a comorbidity	
Developmental delay	36 (57.1)
Dyslexia	5 (7.9)
Intellectual disability	4 (6.3)
ADHD	3 (4.8)
Learning disability	3 (4.8)
Home participation	
Frequency, median (IQR)	4.69 (4.23–5.46)
Involvement, mean ± SD	3.52 ± 0.68
Daycare/preschool participation	
Frequency, median (IQR)	5.00 (3.67–5.33)
Involvement, mean ± SD	3.25 ± 1.14
Community participation	
Frequency, mean ± SD	3.09 ± 0.70
Involvement, mean ± SD	3.41 ± 0.82

* There was one missing value. Abbreviations: SD, standard deviation; ADHD, Attention Deficit Hyperactivity Disorder. IQR, interquartile range.

**Table 2:** Characteristics of the respondents.

Characteristics	Value
Role of respondent, *n* (%)	
Mother	48 (76.2)
Father	15 (23.8)
Respondent’s age (year), mean ± SD	39.07 ± 6.03
Respondent’s education, * *n* (%)	
Junior high school or less	3 (4.8)
Senior high school	34 (54.0)
Diploma/associate degree	5 (7.9)
Undergraduate	16 (25.4)
Postgraduate	4 (6.3)
Monthly family income (in HKD), n (%)	
<20,000	18 (28.6)
≥20,000	45 (71.4)

* There was one missing value. Abbreviations: SD, standard deviation; HKD, Hong Kong dollars.

**Table 3:** Univariate associations between children’s participation and demographical variables in the study.

Settings and Variables	Participation Frequency				Participation Involvement			
	*B*	*95% CI*	*R^2^*	*p*	*B*	*95% CI*	*R^2^*	*p*
**Home setting**								
Presence of comorbidity (yes)	−0.143	−0.355, 0.070	0.029	0.185	−0.380	−0.728, −0.032	0.072	0.033 *
Number of siblings (≥one sibling)	−0.052	−0.265, 0.160	0.004	0.624	0.018	−0.339, 0.375	<0.001	0.919
Monthly family income (≥HKD 20,000)	0.131	−0.094, 0.356	0.022	0.250	0.195	−0.184, 0.573	0.017	0.308
Respondent’s age	−0.023	−0.039, –0.007	0.115	0.006 *	−0.022	−0.051, 0.006	0.039	0.120
Respondent’s education (with university degree)	0.195	−0.017, 0.407	0.053	0.071	0.363	0.009, 0.716	0.064	0.045 *
**Daycare/preschool setting**								
Presence of comorbidity (yes)	−0.091	−0.341, 0.159	0.009	0.467	−0.439	−1.039, 0.161	0.035	0.148
Number of siblings (≥one sibling)	0.070	−0.178, 0.317	0.005	0.576	0.098	−0.503, 0.700	0.002	0.745
Monthly family income (≥HKD 20,000)	0.226	−0.032, 0.484	0.048	0.085	0.534	−0.095, 1.163	0.046	0.095
Respondent’s age	−0.010	−0.030, 0.010	0.017	0.313	−0.017	−0.066, 0.031	0.009	0.474
Respondent’s education (with university degree)	0.331	0.092, 0.571	0.112	0.007 *	0.396	−0.213, 1.004	0.027	0.198
**Community setting**								
Presence of comorbidity (yes)	−0.282	−0.650, 0.086	0.038	0.131	−0.514	−0.933, −0.096	0.091	0.017 *
Number of siblings (≥one sibling)	0.084	−0.286, 0.454	0.003	0.651	−0.314	−0.740, 0.112	0.036	0.145
Monthly family income (≥HKD 20,000)	0.204	−0.189, 0.596	0.018	0.303	0.374	–0.079, 0.828	0.043	0.104
Respondent’s age	−0.018	−0.047, 0.012	0.023	0.237	0.008	–0.027, 0.043	0.004	0.634
Respondent’s education (with university degree)	0.245	−0.129, 0.619	0.028	0.195	0.421	0.010, 0.852	0.060	0.055

Note: Univariate linear regression models were used. Categorical variables are presented with a parenthesis in which the characteristic of interest is specified. * p < 0.05. Abbreviations: *B*, nonstandardized beta parameter estimate; *CI*, confidence interval; *R*^2^, *R* square value; *HKD*, Hong Kong dollars.

**Table 4:** Relationships between parents’ affiliate stigma and children’s participation after controlling for demographic variables in hierarchical regression models.

Participation Variables ^a^	Model Effects		Parents’ Affiliate Stigma						
			Parameter Estimates				Bootstrap		
	*R^2^*	*p*	*β*	*B*	*95% CI*	*p*	*Bias*	*BCa, 95% CI*	*p*
**Home setting**									
Participation frequency ^b^	0.124	0.048 *	−0.102	−0.085	−0.304, 0.134	0.441	−0.009	−0.281, 0.080	0.381
Participation involvement ^c^	0.210	0.008 *	−0.232	−0.325	−0.674, 0.025	0.068	0.004	−0.631, 0.017	0.054
**Daycare/preschool setting**									
Participation frequency ^d^	0.256	0.001 *	−0.058	−0.057	−0.290, 0.176	0.628	0.001	−0.293 0.208	0.641
Participation involvement	0.048	0.236	−0.203	−0.476	−1.110, 0.158	0.138	−0.003	−1.091, 0.227	0.173
**Community setting**									
Participation frequency	0.040	0.296	−0.133	−0.192	−0.583, 0.199	0.330	0.011	−0.530, 0.173	0.240
Participation involvement ^e^	0.254	0.001 *	−0.329	−0.555	−0.963, −0.146	0.009 *	−0.004	−0.928, −0.155	0.008 *

^a^ The child’s age was controlled in all models. ^b^ The respondent’s age was additionally controlled. ^c^ The presence of comorbidity in the child and respondent’s education were additionally controlled. ^d^ The respondent’s education was additionally controlled. ^e^ The presence of comorbidity in the child was additionally controlled. * *p* < 0.05. Abbreviations: *R^2^, R* square value; *β*, standardized beta parameter estimate; *B,* nonstandardized beta parameter estimate; *CI*, confidence interval; *BCa*, bias-corrected accelerated.

**Table 5:** Correlations between the scores of the parents’ affiliate stigma and children’s participation in each activity across three settings.

Participation Items	Spearman’s *ρ* Coefficients (*BCa*, 95% *CI*) ^a^	
	Frequency	Involvement
**Home** **setting**		
Getting rest	0.033 (−0.219, 0.290)	−0.131 (−0.373, 0.101)
Personal care management	0.109 (−0.169, 0.378)	−0.195 (−0.456, 0.055)
Getting clean	0.077 (−0.203, 0.347)	−0.289 * (−0.550, −0.011)
Mealtime	0.107 (−0.162, 0.360)	−0.077 (−0.306, 0.166)
Cleaning up	−0.038 (−0.310, 0.244)	−0.130 (−0.384, 0.159)
Meal preparation	−0.094 (−0.353, 0.167)	0.185 (−0.148, 0.519)
Taking care of other family members	−0.162 (−0.420, 0.122)	0.060 (−0.289, 0.418)
Laundry and dishes	−0.041 (−0.316, 0.237)	0.046 (−0.290, 0.375)
Arts, crafts, stories, music	0.027 (−0.225, 0.228)	−0.132 (−0.376, 0.128)
Screen time	−0.220 (−0.439, 0.022)	−0.014 (−0.298, 0.256)
Indoor play and games	−0.162 (−0.407, 0.091)	−0.073 (−0.312, 0.165)
Celebrations at home	0.031 (−0.247, 0.308)	−0.070 (−0.323, 0.186)
House guests	−0.010 (−0.375, 0.174)	−0.103 (−0.371, 0.176)
**Daycare/preschool** **setting**		
Group learning	−0.003 (−0.270, 0.258)	−0.228 (−0.477, 0.021)
Socializing with friends	0.153 (−0.110, 0.417)	−0.129 (−0.395, 0.124)
Field trips and events	−0.008 (−0.240, 0.227)	−0.084 (−0.339, 0.178)
**Community setting**		
Shopping and errands	−0.074 (−0.325, 0.180)	−0.169 (−0.412, 0.092)
Dinning out	−0.107 (−0.363, 0.161)	−0.236 (−0.506, 0.037)
Routine appointments	0.008 (−0.248, 0.258)	−0.065 (−0.324, 0.208)
Classes and lessons	−0.032 (−0.284, 0.227)	−0.209 (−0.480, 0.111)
Organized physical activities	−0.026 (−0.293, 0.236)	−0.453 ** (−0.676, −0.169)
Community attractions	−0.120 (−0.355, 0.119)	−0.026 (−0.282, 0.232)
Religious/spiritual activities	0.050 (−0.184, 0.281)	0.142 (−0.372, 0.592)
Social gatherings	0.015 (−0.231, 0.258)	−0.193 (−0.441, 0.078)
Community events	−0.064 (−0.306, 0.193)	−0.100 (−0.377, 0.176)
Unstructured physical activities	0.144 (−0.136, 0.404)	0.047 (−0.203, 0.295)
Overnight trips, vacations, and visits	−0.046 (−0.288, 0.209)	−0.160 (−0.435, 0.114)

^a^ The confidence intervals were estimated using Bootstrapping technique. Abbreviations: *BCa*, bias-corrected accelerated; *CI*, confidence interval. * *p* < 0.05.** *p* < 0.01.
